# Effects of antibiotic treatment and phagocyte infiltration on development of *Pseudomonas aeruginosa* biofilm—Insights from the application of a novel PF hydrogel model *in vitro* and *in vivo*

**DOI:** 10.3389/fcimb.2022.826450

**Published:** 2022-07-26

**Authors:** Hong Wu, Lulu Song, Joey Kuok Hoong Yam, Marian Plotkin, Hengzhuang Wang, Morten Rybtke, Dror Seliktar, Theodoros Kofidis, Niels Høiby, Tim Tolker-Nielsen, Zhijun Song, Michael Givskov

**Affiliations:** ^1^ Costerton Biofilm Center, and Institute of International Health, Immunology and Microbiology, Faculty of Health and Medical Sciences, University of Copenhagen, Copenhagen, Denmark; ^2^ Department of Clinical Microbiology, Rigshospitalet, University of Copenhagen, Copenhagen, Denmark; ^3^ The Singapore Centre on Environmental Life Sciences Engineering, Nanyang Technical University, Singapore, Singapore; ^4^ Nanoscience and Nanotechnology Initiative, Faculty of Engineering, National University of Singapore, Singapore, Singapore; ^5^ Department of Biomedical Engineering, Technion-Israel Institute of Technology, Haifa, Israel; ^6^ Department of Surgery, Yong Loo Lin School of Medicine, National University of Singapore, Singapore, Singapore; ^7^ Department of Cardiac, Thoracic and Vascular Surgery, National University Heart Centre, National University Health System, Singapore, Singapore; ^8^ Department of Clinical Microbiology, Hospital South West Jutland, University Hospital of Southern Denmark, Esbjerg, Denmark

**Keywords:** biofilm infection, antibiotic resistance, PF hydrogel, *Pseudomonas aeruginosa*, *in vivo* model

## Abstract

**Background and purpose:**

Bacterial biofilm infections are major health issues as the infections are highly tolerant to antibiotics and host immune defenses. Appropriate biofilm models are important to develop and improve to make progress in future biofilm research. Here, we investigated the ability of PF hydrogel material to facilitate the development and study of *Pseudomonas aeruginosa* biofilms *in vitro* and *in vivo*.

**Methods:**

Wild-type *P. aeruginosa* PAO1 bacteria were embedded in PF hydrogel situated *in vitro* or *in vivo*, and the following aspects were investigated: 1) biofilm development; 2) host immune response and its effect on the bacteria; and 3) efficacy of antibiotic treatment.

**Results:**

Microscopy demonstrated that *P. aeruginosa* developed typical biofilms inside the PF hydrogels *in vitro* and in mouse peritoneal cavities where the PF hydrogels were infiltrated excessively by polymorphonuclear leukocytes (PMNs). The bacteria remained at a level of ~10^6^ colony-forming unit (CFU)/hydrogel for 7 days, indicating that the PMNs could not eradicate the biofilm bacteria. β-Lactam or aminoglycoside mono treatment at 64× minimal inhibitory concentration (MIC) killed all bacteria in day 0 *in vitro* biofilms, but not in day 1 and older biofilms, even at a concentration of 256× MIC. Combination treatment with the antibiotics at 256× MIC completely killed the bacteria in day 1 *in vitro* biofilms, and combination treatment in most of the cases showed significantly better bactericidal effects than monotherapies. However, in the case of the established *in vivo* biofilms, the mono and combination antibiotic treatments did not efficiently kill the bacteria.

**Conclusion:**

Our results indicate that the bacteria formed typical biofilms in PF hydrogel *in vitro* and *in vivo* and that the biofilm bacteria were tolerant against antibiotics and host immunity. The PF hydrogel biofilm model is simple and easy to fabricate and highly reproducible with various application possibilities. We conclude that the PF hydrogel biofilm model is a new platform that will facilitate progress in future biofilm investigations, as well as studies of the efficacy of new potential medicine against biofilm infections.

## Introduction

*Pseudomonas aeruginosa* is an important opportunistic pathogen responsible for a substantial part of the refractory nosocomial infections (Høiby et al., 2001). In the majority of the problematic *P. aeruginosa* infections, the bacteria are present in the form of biofilms (Tolker-Nielsen, 2014; Hentzer et al., 2003). Bacterial biofilms can be described as aggregated cells embedded in self-produced extracellular polymeric substances. Biofilms are causing persistent infections, e.g., when they are present on implanted material or are present as bacterial aggregates embedded in lung mucus or wound material (Rybtke et al., 2015).

Common clinical biofilm infections include implant or prosthesis-related infections, lung infections in cystic fibrosis patients, and infections in burn patients (Lynch and Robertson, 2008; Høiby et al., 2010a; Song et al., 2013; Wu et al., 2015). The characteristic feature of biofilm infections is an enhanced tolerance to antimicrobials, which makes eradication of the infections almost impossible by conventional antibiotic therapies and host immune defences (Høiby et al., 2010; Wu et al., 2015; Ciofu and Tolker-Nielsen, 2019).

Various biofilm models have been developed and used in biofilm research for at least three decades, including *in vitro* models (static and dynamic systems), *ex vivo* models (tissue and cell cultures), and *in vivo* models (invertebrate or vertebrate animal models) (Lebeaux et al., 2013; Rybtke et al., 2015). However, the lack of chronic features in these biofilm models has long hindered progress in biofilm research. It is essential that the study model exhibits the typical features and symptoms of real chronic infections (Costerton et al., 2003). Clinical outcomes of routine antibiotic susceptibility tests currently correlate poorly with antibiotic treatment outcomes, which may indicate a need for improved assessment platforms. After investigating different materials and substances for their ability to support bacterial biofilm formation, PF hydrogel was selected as an outstanding candidate and used in our biofilm studies. PF hydrogel with cell-compatible features has become a popular synthetic biomaterial used as temporary tissue scaffold and drug delivery vehicle in biomedical sciences (Seliktar, 2012). PF hydrogel is a polymeric material containing plenty of water and can be polymerized together with live cells and/or therapeutic compounds. Importantly, PF hydrogels have been shown to provide cells with a three-dimensional scaffold, thereby enhancing cell survival and cell proliferation (Tibbitt and Anseth, 2009).

Because of the potential advantages of biofilm models built with hydrogel, we designed the present study to investigate systemically the development of bacterial biofilms in an optimized model and to investigate the effects of phagocytes and antibiotics in mono or combination therapy on *P. aeruginosa* biofilm infections.

## Materials and methods

### Bacterial strains, antibiotics, media, and culture conditions

The bacterial strain used in the present study was *P. aeruginosa* PAO1 (kindly provided by Professor Iglewski, University of Rochester). The antibiotics used in the study were imipenem (β-lactam) (Tienam^®^ imipenem/cilastatin, MSD) and/or gentamicin (aminoglycoside) (Hexamycin^®^, Sandoz A/S, Denmark). Luria–Bertani (LB) and a growth minimal medium called. ABTG (Clark and Maaløe, 1967) media were used for culture of *P. aeruginosa* at 37°C.

### Preparation of PF hydrogel biofilm implants

For details on the synthesis of PF hydrogels, please refer to previous publications (Dikovsky et al., 2006; Dikovsky et al., 2008 and Elbert and Hubbell, 2001). The final PF hydrogel product was **PEG-DA** moieties attached to a cysteine residue on the **Fibrinogen** backbone, hereafter named as **PF hydrogel.** The PF hydrogel mixture was made by 0.1% photoinitiator, 2% PEG-DA (w/v) plus PF hydrogel (8 mg/ml), and LB with/without *P. aeruginosa* cells. The mixture of 100 μl was pipetted into the wells of microplate and polymerized under UV light at 365 nm and 5 mW/cm^2^ (VL-6.L 365 nm 1 × 6 W Lamp), and it was demonstrated that UV energy of 5 mW/cm^2^ did not reduce bacterial number or interfere with bacterial viability.

### Establishment of biofilm *in vitro* and *in vivo*

Twenty-five microliter of ABTG was added to each well containing PF hydrogel (0.1 ml) and cultured at 37°C for 0, 1, 3, and 7 days to enable biofilm formation *in vitro*. All *in vitro* samples for bacteriological studies were in triplicates. For the *in vivo* biofilms, 8- to 9-week-old BALB/cJ mice (Taconic M&B A/S, Denmark or InVivos Pte Ltd., Singapore) were used, and each individual group contained 10 mice. All animal experiments were authorized by the National Animal Ethics Committee (2012-15-2934-0677), Denmark, or ARF SBS/NIE NTU Institutional Animal Care and Use Committee (A-0191 AZ), Singapore. An incision in the left and/or right groin of the mice was made after anaesthesia to expose the peritoneal cavity. The PF hydrogel implants with or without *P. aeruginosa* PAO1 were placed into the right and/or left peritoneal cavity of each mouse. The incisions were closed with a suture and healed without complications.

### Evaluation of bacteriology in *P. aeruginosa* biofilms *in vitro* and *in vivo*

A moisture swab was taken from the peritoneal cavity before acquiring the implant and spleen from the mouse, and the swab was put into a tube containing 1 ml of sterile saline for quantification of the bacteria in the peritoneal cavity. PF hydrogel implants and spleens were collected from wells (*in vitro*) or from peritoneal cavities (*in vivo)* into sterile tubes with 3-ml cold sterile saline. The PF hydrogel implants and spleens were homogenized separately, and all samples were serially diluted and plated on agar plates for determination of bacterial CFU after 24–48 h incubation at 37°C.

### Histological evaluation of biofilms in PF hydrogels

The PF hydrogel implants with or without *P. aeruginosa* were embedded in Tissue-Tek and frozen at −20°C to −40°C. Frozen sections (minimum of 50 μm) of the PF hydrogels were made by using a freeze microtome with Cryostat (Leica CM3050S) and were evaluated directly or with live/dead staining (LIVE/DEAD^®^ *Bac*Light™) using a confocal laser scanning microscope (CLSM) (LSM 780, Carl Zeiss) equipped with a laser detector and filter set for monitoring green fluorescence (excitation, 488 nm; emission, 517 nm). Simulated epifluorescence images were generated with an IMARIS software package (Bitplane AG). Additional frozen sections (ca. 8–10 μm) were stained with Methylene Blue and evaluated by light microscopy using an Olympus BX40-DP71 microscope.

### Efficacy of antibiotics on *P. aeruginosa* biofilms *in vitro* and *in vivo*

The minimal inhibitory concentration (MIC) was 1.25 μg/ml for imipenem/cilastatin (IMP) and 0.5 μg/ml for gentamicin (GM) against planktonic *P. aeruginosa* PAO1. IMP and GM at final concentrations of 64×, 128×, and 256× MIC in ABTG were used in the *in vitro* study for single or combination treatments. The concentration of IMP and GM used in the *in vivo* study was 64× MIC for day 0 infections and 128× MIC for longer infections. The treatment was given by intraperitoneal injection, one dose per day for 2 days, and the time points of assessment started at 4 h after the second treatment.

### Statistical analyses

The significance of the differences of bacterial CFUs and bactericidal percentages between the groups were analyzed by using ANOVA (analysis of variance) and Student’s t-test. *P* < 0.05 was considered statistically significant. The graphs were generated by using a Microsoft Office Excel program.

## Results

### Development of *P. aeruginosa* Biofilms in PF hydrogel *in vitro* and *in vivo*

**Figure 1:**
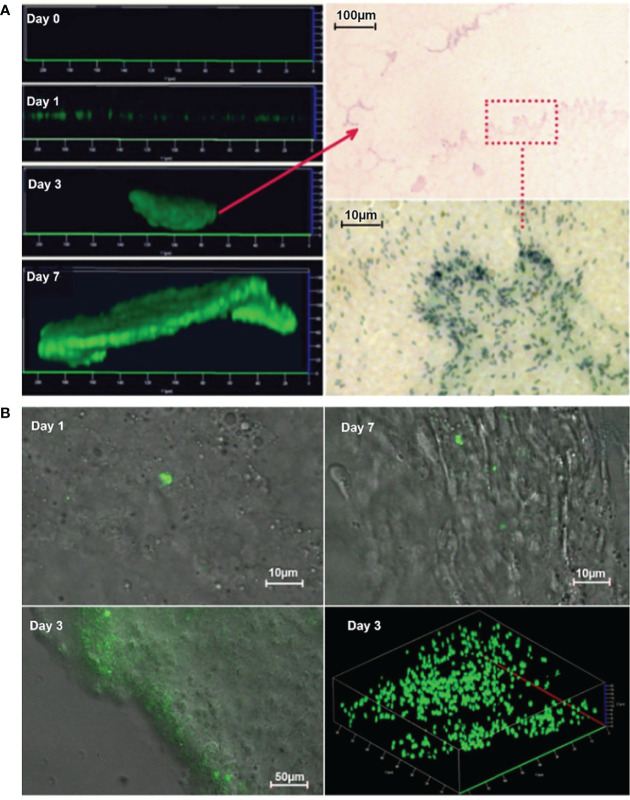
Development of *P. aeruginosa* biofilm in PF hydrogel *in vitro* **(A)** and *in vivo* (**B**). **(A)** Left panel: CLSM micrographs visualize clearly that *P. aeruginosa* PAO1 (labeled with GFP) develops biofilms progressively from single bacteria (almost no signal due to limited number of bacteria) at day 0, further to microcolonies at day 1 that increased in size at days 3 and 7. Right panel: Images taken by light microscopy of methylene stained day 3 biofilms show distinct biofilm structures and single bacterial cells inside the PF hydrogel. The red arrow points toward a microcolony in the upper image and the bacteria in the red rectangle area are visualized by higher magnification in the lower image. **(B)** Epifluorescence and merged epifluorescence/light images acquired in frozen sections of PF hydrogel implants harvested from mouse peritoneal cavities. PAO1 (labeled with GFP) formed first single microcolonies at day 1 and then developed biofilm in an extensive area at day 3.

*P. aeruginosa* PAO1 tagged with green fluorescent protein (GFP) was demonstrated to form typical biofilms in PF hydrogels *in vitro* and *in vivo* (**Figure 1**). For the *in vitro* PF hydrogels, CLSM images visualized clearly that *P. aeruginosa* developed biofilms progressively from single bacteria at day 0, further to microcolonies at day 1, that increased in size at days 3 and 7 (**Figure 1A** **, left panel**). The biofilm present at day 3 was histologically evaluated, and distinct biofilm structures and single bacterial cells inside the PF hydrogel were detected (**Figure 1A** **, right panel**). For the frozen sections of PF hydrogel implants harvested from mouse peritoneal cavities, light and CLSM microscopy showed single microcolonies at day 1, and biofilm in an extensive area inside the PF hydrogel at day 3, which indicated the PF hydrogel, could be first a shelter for bacteria from host immunity and then a good substance for bacteria to form biofilm in *in vivo* (**Figure 1B**). It was expected to see extensive green fluorescence emitted from the biofilm bacteria *in vivo* on day 7, consistent with the large number of bacteria detected in the PF hydrogel samples. However, this was not the case, only a few green signals were discovered (**Figure 1B**). Most bacteria did not emit green light, possibly due to consumption of molecular oxygen by the surrounding large numbers of phagocytes, as oxygen is required for the expression of the fluorophore in GFP (Heim et al., 1994).

### Bacterial quantification in *P. aeruginosa* peritoneal biofilm infections in mice

**Figure 2:**
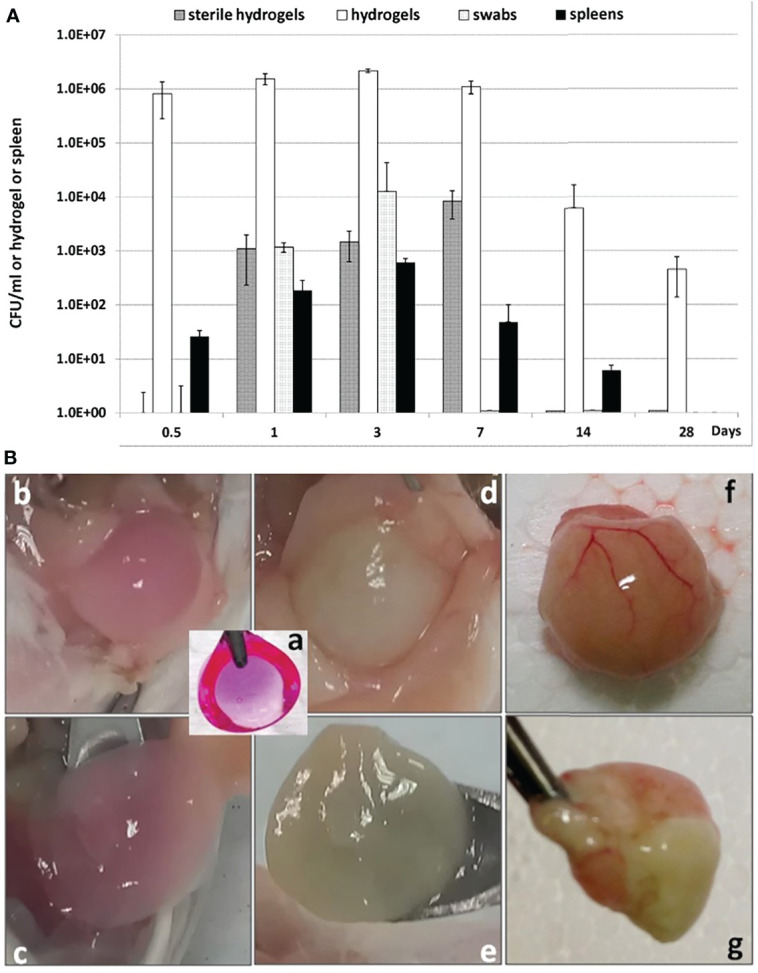
Quantification of *P. aeruginosa* bacteria present in PF hydrogels harvested from mouse peritoneal cavities **(A)** and visualization of the harvested PF hydrogels (**B**). **(A)** Enumeration of the number of bacteria in the infections. All bar charts show the median ± standard deviation (n ≥ 6). Values are shown for bacteria in hydrogels (white bars), spleens (black bars), originally sterile hydrogels (dark gray bars), and peritoneal swabs (light gray-bars). **(B)** Gross view of PF hydrogels with or without *P. aeruginosa* harvested from mouse peritoneal cavities. **(a)** PF hydrogel implant before implantation (color induced by Resazurin sodium); **(b, c)** sterile PF hydrogel on day 3 after implantation was generally loose and easily collected from the mouse peritoneal cavity; **(d, e)** PF hydrogel with *P. aeruginosa* on day 3 after implantation was wrapped by peritoneal omentum and could still be carefully separated; **(f, g)** PF hydrogel with *P. aeruginosa* on day 14 after implantation was wrapped tightly by the peritoneal omentum with pus inside, and was impossible to separate from the peritoneum.

For the *in vivo* PF hydrogels, the bacterial density increased from the inoculum with approximately 10^3^ CFU per hydrogel to approximately 10^6^ CFU per hydrogel within 12 h after inoculation and remained constant at least for 7 days. Moreover, *P. aeruginosa* could be detected in the PF hydrogels on days 14 and 28 after inoculation (**Figure 2A**), indicating that the PF hydrogel facilitated a long-term bacterial biofilm infection in the mouse peritoneal cavities. The results were highly reproducible regardless the variation of the inoculum between ~10^2^ and ~10^4^ CFU per hydrogel. Bacteria could be detected in the peritoneal cavities for only 3 days, indicating a transient acute peritonitis. Bacteria were also detected in PF hydrogels that were fabricated without bacteria (sterile hydrogels) and placed in the abdomen together with the bacteria-containing hydrogels. This indicated that the PF hydrogels could be colonized by bacteria from the peritoneal cavity *in vivo*. Bacteria were detected in the spleen for as long as the existence of the peritoneal biofilm infection, which reflects dissemination of *P. aeruginosa* from the peritoneal biofilm into the blood stream (**Figure 2A**). Nevertheless, the bacteria in the spleens were relatively low in number, approximately 10^3^ CFU per spleen, which might suggest that the animals suffered from only slight bacteraemia, but not sepsis, as the animals exhibited no symptoms and seemed healthy throughout the experiment.

### Infiltration of phagocytes in *P. aeruginosa* biofilms

**Figure 3:**
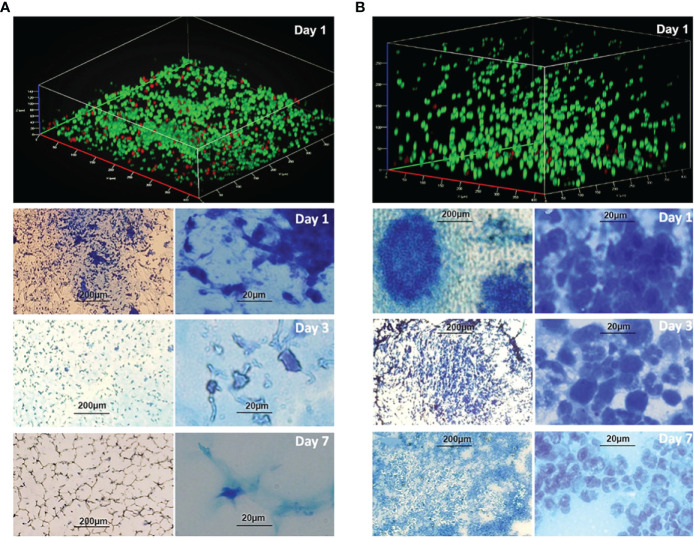
Infiltration of phagocytes in the PF hydrogels without **(A)** and with **(B)** *P. aeruginosa* PAO1 *in vivo.* Images were acquired in PF hydrogels harvested from mouse peritoneal cavities at days 1, 3, and 7 after implantation and were stained with live/dead stain or methylene blue. **(A)** Upper image: CLSM micrograph of live/dead stained sterile PF hydrogel indicates that it was penetrated by a thin layer (ca. 40 µm) of phagocytes. Lower images: Infiltration of phagocytes in the sterile PF hydrogels visualized by light microscopy at different magnifications (bar sizes: left, 200 µm; right, 20 µm). The phagocytes in the PF hydrogels were mostly peritoneal macrophages, and the infiltrations were remarkable only at day 1, reduced significantly at day 3, and disappeared completely at day 7. **(B)** Upper image: CLSM micrograph of live/dead stained bacteria-containing PF hydrogel indicates that it is penetrated by a thick layer (> 250 µm) of phagocytes. Lower images: Infiltration of phagocytes in the PF hydrogels visualized by light microscopy at different magnifications (bar sizes: left, 200 µm; right, 20 µm). The images show a high density of host immune cells in the PF hydrogels predominated by PMNs, and the infiltration of PMNs was continuously observed in the whole study period. PMNs migrated toward and stayed close to the bacterial biofilms. The PMNs dispersed widely following the development of biofilm at days 3 and 7.

Phagocyte infiltrations were observed in the peritoneal PF hydrogel implants with or without *P. aeruginosa*. The infiltrations differed with respect to the type of phagocytes and the duration of infiltration (**Figure 3**). The sterile PF hydrogel implants were mainly infiltrated by a thin layer (40 µm) of peritoneal macrophages by day 1, which was significantly reduced by day 3 and completely gone by day 7 (**Figure 3A**). In contrast, a thick layer (250 µm) of polymorphonuclear leukocytes (PMNs) dominated the infiltration in the PF hydrogels with *P. aeruginosa* in the entire study period (**Figure 3B**). The PMNs migrated close to the biofilms but could not clear them efficiently as the bacterial CFU continuously was at a level of ~10^6^ CFU per PF hydrogel (**Figure 2A**). On day 14 after intraperitoneal challenge, the hydrogels inoculated with P. aeruginosa were found tightly wrapped by the greater omentum in the mouse abdominal cavity, and a large abscess formed (**Figure 2B**). Strong and durable host immune responses were observed only in the PF hydrogels inoculated with *P aeruginosa*, in contrast to the sterile PF hydrogels that induced only mild transient inflammatory responses.

### Evaluation of antibiotic treatments of biofilms *in vitro* and *in vivo*

**Figure 4:**
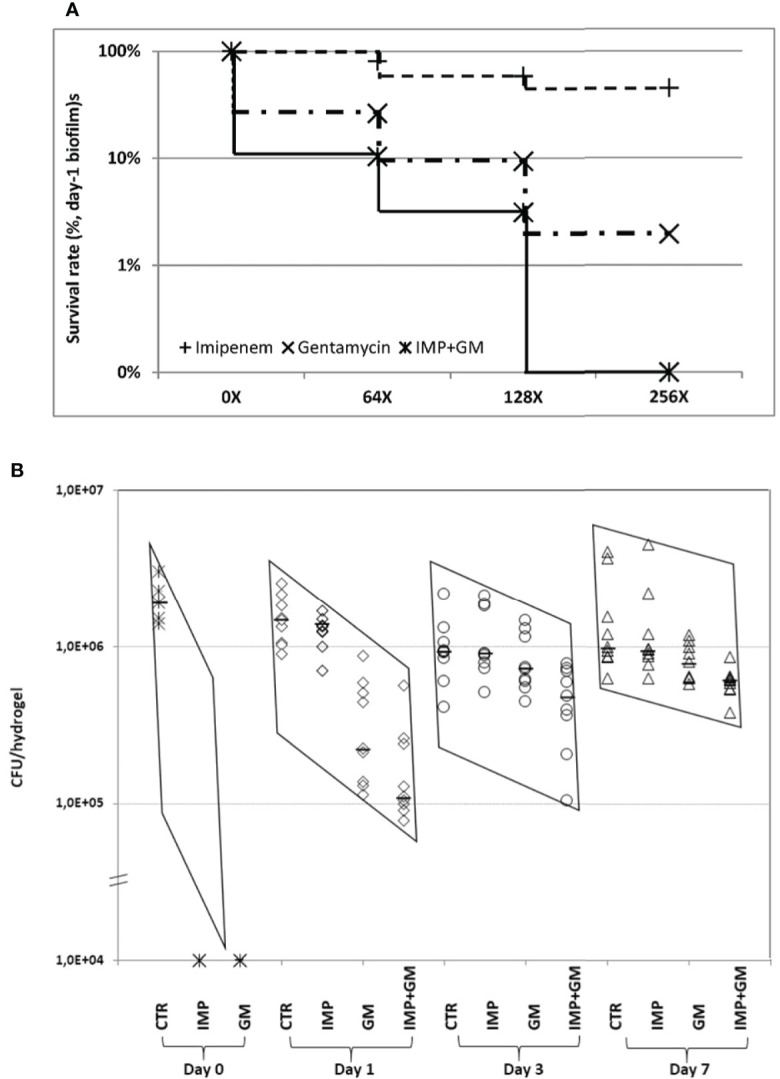
Evaluation of mono and combination therapies of β-lactam (imipenem, IMP) and/or aminoglycoside (gentamycin, GM) in *P. aeruginosa* PAO1 biofilms *in vitro* **(A)** and *in vivo* (**B**). **(A)** Comparing the doses of 0×, 64×, 128×, and 256× MIC in monotherapy or combination therapy, it appears that higher concentrations of antibiotics exhibited stronger killing against the biofilm bacteria. However, significant difference was only seen in the combination treatment, *p* < 0.02. Increasing the dose from 64× to 128× MIC and 128× to 256× MIC in monotherapy did not show significant differences. **(B)** Bacteria in day 0 biofilms were 100% killed by IMP or GM alone at 64× MIC, which is consistent with the *in vitro* findings. In contrast, IMP monotherapy at 128× MIC killed <10% and <3% of the bacteria in day 1, 3, and 7 biofilms, respectively. GM alone at 128× MIC killed 85% of the biofilm bacteria and exhibited better bactericidal effect than IMP (*p* < 0.0001) at day 1 biofilms. Combination treatment at the same MIC killed 92.5%, 47.5%, and 38.5% of the bacteria in day 1, 3, and 7 biofilms, respectively, in which, killing rates were significantly higher than for either antibiotic monotherapy at all assessed time points (*p* < 0.03). The two sharply sloped parallelograms of the data distribution indicate that antibiotic treatment was more effective in young biofilm infections (days 0 and 1) compared with mature biofilm infections (days 3 and 7). CTR, control; IMP, imipenem; GM, gentamicin; IMP + GM, imipenem + gentamicin combination. MICs: IMP, 1.25 μg/ml; and GM, 0.5 μg/ml.

Antibiotic treatment at different ages of *P. aeruginosa* biofilms showed that imipenem (IMP) or gentamicin (GM) monotherapy at 64× MIC killed 99.9% of the bacteria merely in day 0 biofilms and eliminated less than 90% of the bacteria in day 1 and older biofilms (**Figure 4A**), which suggests the importance of early antibiotic treatment. Combination therapy with IMP and GM at 64× MICs improved significantly the bactericidal effect in day 1 and 3 biofilms, indicating that combination therapy worked more efficient than monotherapy. However, it was not possible to eliminate more than 95% of the bacteria in day 1 or older biofilms (**Table 1**). The killing of the biofilm bacteria was concentration-dependent, i.e., higher doses had better bactericidal effects in both mono and combination therapies. Nevertheless, the bactericidal differences between higher and lower doses were only significant in the combination treatments, in which the IMP-GM combination at 256× MIC eliminated more than 99.9% of the bacteria in day 1 biofilms (**Table 2** and **Figure 4A**), implicating that both combination therapy and high dosage are key points for elimination of biofilm infections. Extension of antibiotic treatments from 4 to 8 h increased the bactericidal effects of the antibiotics in relatively mature biofilms (**Table 3**). Hence, in addition to concentration-dependent killing, time-dependent killing was also observed in the biofilm treatments. For the *in vivo* biofilm infections of *P. aeruginosa* established in mouse peritoneal cavities, IMP or GM monotherapy killed completely day 0 biofilm bacteria in accordance with the *in vitro* study. However, mono and combination therapies at 128× MIC (IMP, 160 mg/kg; GM, 64mg/kg) killed less bacteria in day 1 and older biofilms (**Table 4** and **Figure 4B**) compared with the *in vitro* study (**Table 2**). The combination therapies killed less than 50% of the bacteria in day 3 and 7 biofilms, exhibiting poor outcomes *in vivo*; however, the poor killing rates were still significantly better than the killing rates observed for monotherapies (**Figure 4B**).

**Table 1:** Efficacies of antibiotics on bactericidal rate (%) at different ages of *P. aeruginosa* biofilms (64× MIC, 4-h treatments *in vitro*, mean ± SE).

Ages of Biofilms	Imipenem (IMP)	Gentamicin (GM)	IMP + GM
**Day 0**	99.96 ± 0.01	>99.99 ± 0.00	>99.99 ± 0.00
**Day 1**	22.59 ± 11.55^¤^	87.05 ± 1.03^#^	94.25 ± 0.81*
**Day 3**	10.20 ± 18.44^¤^	41.16 ± 15.55^#^	82.85 ± 2.17**

The combination therapy improves significantly the bactericidal effects at day 1 and day 3 biofilms, compared with IMP or GM monotherapy, *p < 0.02 and **p < 0.001; ^#^ GM mono compared with IMP mono, p < 0.005.

**Table 2:** Effects of different antibiotic doses on biofilm bacteria (bactericidal rate %, day 1 biofilms, 4-h treatments *in vitro*, mean ± SE).

MICs	Imipenem (IMP)^#^	Gentamicin (GM)^#^	IMP + GM*
**0×**	0.00	0.00	0.00
**64×**	0.00	35.30 ± 3.19	49.25 ± 15.94
**128×**	76.67 ± 3.18	93.15 ± 3.55	97.31 ± 1.31
**256×**	88.97 ± 1.39	96.93 ± 1.68	>99.99 ± 0.00

Comparison of the differences between higher and lower MICs (256× vs. 128× and 128× vs. 64×). * p < 0.02 in combination treatments. ^#^ No significance in IMP or GM monotherapy.

**Table 3:** Comparison of treatment time in bactericidal effects on biofilm bacteria (bactericidal rate %; day 3 biofilms; 64× MIC *in vitro*, mean ± SE).

Time	Imipenem (IMP)	Gentamicin (GM)	IMP+GM	*p*-value*
**4 h**	10.20 ± 18.44	41.16 ± 15.55	82.85 ± 2.17	≤0.005
**8 h**	29.30 ± 16.92	70.62 ± 4.01	96.19 ± 0.85	<0.001
***p*-value^#^**	n.s.	≤0.03	<0.02	–

*Combination therapy vs. IMP or GM monotherapy.

^#^8-h vs. 4-h treatments.

n.s., no significance.

**Table 4:** Mono versus combination therapy of antibiotics against biofilm infections (bactericidal rate; 128× MIC; IMP: 160 mg/kg and GM: 64 mg/kg; *in vivo*).

Time	Non-Treatment	Imipenem (IMP)*	Gentamicin (GM)	IMP + GM^#^
**Day 1**	0.00	8.78%	**84.8%^&^**	92.52%
**Day 3**	0.00	2.95%	22.3%	47.54%
**Day 7**	0.00	2.68%	19.1%	38.48%

*IMP mono compared to non-treatment, no significance. ^&^GM compared with IMP and non-treatment at day 1 after infection, p < 0.0001. ^#^Combination compared to non- or monotherapy of IMP or GM, p < 0.03.

## Discussion

Biofilm studies have been established by means of bacteria embedded in agar or seaweed alginate or pre-attached to silicone implants, and these manipulations were shown to protect the bacteria from host immune defences and antibiotics (Christensen et al., 2007; Hengzhuang et al., 2012). However, these models possess several limitations, and the most critical drawback is the high inoculum of 10^7^–10^8^ CFU required to initiate an *in vivo* infection, because animals with normal immunity are not susceptible to planktonic *P. aeruginosa* (Moser et al., 1997; Christensen et al., 2007). The accumulation of lipopolysaccharide endotoxin released from the high inoculum of bacteria unavoidably induced severe acute symptoms, such as weight loss, bleeding, dehydration, fever or hypothermia, and even mortality (Rietschel and Brade, 1992; Moser et al., 1997).

Therefore, we appreciate the present biofilm model facilitated by PF hydrogel, which provides a real developing biofilm infection *in vivo* rather than the prebuilt biofilms transferred directly into animals. This biofilm model also has the advantage of the typical chronic features, such as a persistent large quantity of bacteria in the infected foci, continuous mild bacteraemia for more than 2 weeks, and the hosts in general with a “healthy” appearance including stable body weight with normal body temperature and normal appetite and activity. Moreover, an important feature of the PF hydrogel is biocompatibility with bacteria (**Figure 1**) and host phagocytes (**Figure 3**), which is demonstrated by its use in human medicine for years (Slaughter et al., 2009). Therefore, we pointed out that our PF hydrogel biofilm model is a platform that will facilitate the progress in microbial biofilm studies, especially *in vivo*.

Biofilm infections are known to be tolerant to immune defenses. Our results confirmed that PMN phagocytes could migrate and distribute all over the bacterial biofilms (**Figure 3** **, right**), but it appeared that the PMNs were not able to kill the biofilm bacteria. The reason might be that the aggregated *P. aeruginosa* in the biofilm inhibited the phagocytosis of PMNs. The biofilm matrix and large size of the aggregates may provide protection against phagocytosis (Moser et al., 2021). Furthermore, Jensen et al. reported that the oxidative burst response induced by biofilm bacteria in PMNs was significantly slower and weaker than that of planktonic bacteria (Jensen et al., 1990). It was demonstrated that rhamnolipids, products of the *rhlA* gene in *P. aeruginosa*, efficiently induce PMN lysis (Jensen et al., 2007), and inactivation of the *rhlA* gene disabled bacterial protection against PMNs (Van Gennip et al., 2009).

Studies evaluating the efficiency of antibiotic mono or combination treatment against biofilm infections are important and valued by the medical community. However, only a few studies have performed such research in models mimicking real biofilm infections. Aminoglycoside in combination with β-lactam antibiotics is often used intravenously in hospitals as the major treatments against *P. aeruginosa* infection (Ramsey, 1996). Comparison of *P. aeruginosa* isolates growing as planktonic cells, adherent monolayer, and biofilms showed that the susceptibility to mono treatment with β-lactam, aminoglycoside, and fluoroquinolone was decreasing in the order planktonic cells, adherent monolayer, and biofilm (Aaron et al., 2002). Furthermore, biofilm bacteria, but not adherent bacteria, were significantly more resistant to two- or three-antibiotic combination treatments than the planktonic bacteria (Aaron et al., 2002). Our *in vivo* results are in accordance with these previous *in vitro* findings, because day 0 biofilms (**Figure 4B**), similar to adherent cells, could be easily cleared by β-lactam or aminoglycoside mono treatment. Our study further demonstrated that combination therapy at ≥256× MICs was able to eliminate 99.9% of the bacteria in day 1 biofilms (young biofilms), but not in day 3 and day 7 biofilms (mature biofilms), emphasizing that early antibiotic treatment is essential to fully eradicate biofilm infections (**Tables 1** and **4**, **Figure 4B**). In general, concentration-dependent killing was demonstrated in our study (**Table 2**). It was impossible to remove more than 50% of the bacteria in mature *in vivo* biofilms with combination treatment employing β-lactam and aminoglycoside antibiotic at 128× MICs (**Table 4**, **Figure 4B**), which further demonstrated that once the biofilm infection is established, it is impossible to eradicate the infection by antibiotic treatment alone. Smith et al. reported that combination treatment with β-lactam and aminoglycoside antibiotic produced a longer clinical remission without infection symptoms than β-lactam mono treatment on the management of a pulmonary exacerbation in cystic fibrosis patients (Smith et al., 1999). Herrmann et al. found that combination treatment of colistin and tobramycin showed significantly better bactericidal effects on in vitro P. aeruginosa biofilms compared with the monotherapies (Herrmann et al., 2010). On the other hand, combination antibiotic treatment helps to reduce the risk of antibiotic resistance according to the ESCMID (European Society of Clinical Microbiology and Infectious Diseases) guidelines for the diagnosis and treatment of biofilm infections (Høiby et al., 2015). Therefore, we conclude that combination therapy with high doses of antibiotics potentially can be used to control (but not eradicate) biofilm infections in the clinic.

## Conclusions

In conclusion, we have developed a PF hydrogel–based biofilm model that has many of the features of real biofilm infections, including progressive biofilm development, chronicity, immune response, and antibiotic tolerance. We pointed out that that this biofilm model will be useful for future biofilm research, as well as studies of the efficiency of new potential medicine against biofilm infections.

## Data availability statement

The original contributions presented in the study are included in the article/supplementary material. Further inquiries can be directed to the corresponding author.

## Ethics statement

The animal study was reviewed and approved by The National Animal Ethics Committee (2012-15-2934-0677), Denmark and ARF SBS/NIE NTU Institutional Animal Care and Use Committee (A-0191 AZ), Singapore.

## Author contributions

Study design: HW, ZS, and MG. Major researchers: LS, HW, ZS, JH, HZW, and MP. Figures, tables, and statistical analyses: HW. Manuscript writing and revision: HW, ZS, MG, NH, and TT-N. Project guidance: MG, NH, and TT-N. Partly support of research materials: MR, DS, and TK. Financial support: MG, TK, and NH. All authors contributed to the article and approved the submitted version.

## Funding

The study was supported by the Danish Strategic Research Council to MG (CAR) and the National Research Foundation - Technion - National University of Singapore (R-398-000-065-592) to the Regenerative Medicine Initiative in Cardiac Restoration Therapy.

## Acknowledgments

The study was supported by the Danish Strategic Research Council to MG (CAR) and the National Research Foundation - Technion - National University of Singapore (R-398-000-065-592) to the Regenerative Medicine Initiative in Cardiac Restoration Therapy. We greatly appreciate the financial support for our research from the two foundations.

We sincerely thank Dr. Lim Lee Wei, Division of Molecular Genetics and Cell Biology, School of Biological Sciences, NTU, for his kind guidance in relation to our histological work.

## Conflict of interest

The authors declare that the research was conducted in the absence of any commercial or financial relationships that could be construed as a potential conflict of interest.

## Publisher’s note

All claims expressed in this article are solely those of the authors and do not necessarily represent those of their affiliated organizations, or those of the publisher, the editors and the reviewers. Any product that may be evaluated in this article, or claim that may be made by its manufacturer, is not guaranteed or endorsed by the publisher.
